# Supplementary material for: Patterns of Intron Gain and Loss in Fungi
Source: PLoS Biol. 2004 Nov 30;2(12):e422. doi: 10.1371/journal.pbio.0020422 (PMC532390; doi:10.1371/journal.pbio.0020422)
Supplement: Table S1 — Also available at http://genes.mit.edu/NielsenEtAl/. (4.3 MB ZIP). [file pbio.0020422.st001.zip › NielsenEtAl/html/1348.html]

AN0432.1.NCU03112.1.MG03708.1.FG00926.1


```
 CLUSTAL W (1.82) Multiple Sequence Alignments - Introns Inserted


Sequence 1: AN0432.1	322 aa
Sequence 2: FG00926.1	331 aa
Sequence 3: NCU03112.1	343 aa
Sequence 4: MG03708.1	331 aa
Alignment Length: 347 aa
Number Identitical Residues: 160 aa
Alignment Score (without introns) 8161


MG03708.1 	-----MFARPAIRACQSLK~Q0---PVRRYTNTPPPQSGNANNSRVGILIASAVGMAGFG
NCU03112.1	MSLFVASTRSAFRAAAPIK~R0SFQTRRSYATEPS----KGGSSSTILLGAAAVGLAGAG
FG00926.1 	-----MFARSAFRAAQPLR0-~---SVRRYATEAG----GAGGSNALLYAAGAAALGGAG
AN0432.1  	-----MFSRYAFRCAQPLR~Q0---SARQYSTEAP----KSKSLAPVYVAVGLAGLG-VG
          	       :* *:*.. .::      . * *:. .      . .       .. ..:.  *

MG03708.1 	TYFMFGQGTTP--AAGVK-----ALGAEPKKALEM-EKGFVSLQLDDVEVVNHNTKRLRF
NCU03112.1	AYFFSGAGAAKKAEASVKQVTEKITPGEIKKAFVGGDQGWLSLKLEEVELVNHNTKRLRF
FG00926.1 	YWYFGKSGAP---VAAAAQDVKQAVGVEPKAAFTGGDQGWVSLKLSDVEIVNHNTKRLRF
AN0432.1  	LYRY-QSGAA---TAEAP--------AERPKVFTGGDQGWVNLKLSDIEILSHNTKRLRF
          	 :     *:.    * .          *   .:  .::*::.*:*.::*::.********

MG03708.1 	KLPEDDMVSGLHVAS~ALLTKFKPEGAEKPVLRPYTPISDED~QKGYLDLIVKKYEGGPM
NCU03112.1	RLPEDDMVSGLHVAS~AILTKFKPIDAEKAVLRPYTPISDES1AQGYIDLLVKKYEGGPM
FG00926.1 	ELPEADQVSGLHVAS~ALLTKYKGPNDEKATLRPYTPISDET1DKGFLDLLIKKYPNGPM
AN0432.1  	EFPDKEAVSGLHIAS1ALLTKYSPPDGSKPVIRPYTPTSDED1QPGYLELVVKRYPNGPM
          	.:*: : *****:** *:***:.  . .*..:***** ***    *:::*::*:* .***

MG03708.1 	STHIHELVPGQKLDFKGPLPKYEWSANK1HPHVAMIAGGTGITPMYQIMRAIFKNPADKT
NCU03112.1	STYLHDMAPGQRLDIKGPLPKYPWEANK~HKHIALVAGGTGITPMYQLIRAIFNNPDDKT
FG00926.1 	STHLHDMAPGQRLDLKGPLPKYAWEENK~HDHIALIAGGTGITPMYQLARAIFNNPKDKT
AN0432.1  	SEHLHNMNVDQRLDFKGPLPKYPWEANK~HKHICLVAGGTGITPMYQLAREIFKNPEDKT
          	* ::*::  .*:**:******* *. ** * *:.::***********: * **:** ***

MG03708.1 	KVTLVVGNITEEDILLKKQLAELENTYPQRFRAFYVLDNPPKDWAGTKGYITKDLLKTVL
NCU03112.1	KVTLVFGNVSEEDVLLKHELATIENHYPQRFRAFYVLDNPPKEWAGNKGYINKDLLKTVL
FG00926.1 	KVTLVFGNVSEQDILLKKQFEELENTYPQRFRAFYVLDNPPKGWAGGSGFISKDLLKTVL
AN0432.1  	KVTLVFGNVSEEDILLKREFEDLENTYPQRFRAFYVLDNPPEGWTGGKGYITKELLKTVL
          	*****.**::*:*:***:::  :** ***************: *:* .*:*.*:******

MG03708.1 	PEPKEENIKVFVCGPPGMMKAISGNKVSPKDQGEVSGILKELGYKQDQIYKF
NCU03112.1	PEPKNEDIKIFVCGPPGMMNSISGNKKSPRDQGELTGILKELGYSPDQVYKF
FG00926.1 	PEPKNENIKLFVCGPPGLMNAISGNKVSPKNQGELTGALKELGYKEDQVYKF
AN0432.1  	PEPKEENIKIFVCGPPGMYKAISGPKVSPKDQGELTGILKELGYSKDQVYKF
          	****:*:**:*******: ::*** * **::***::* ******. **:***
```
